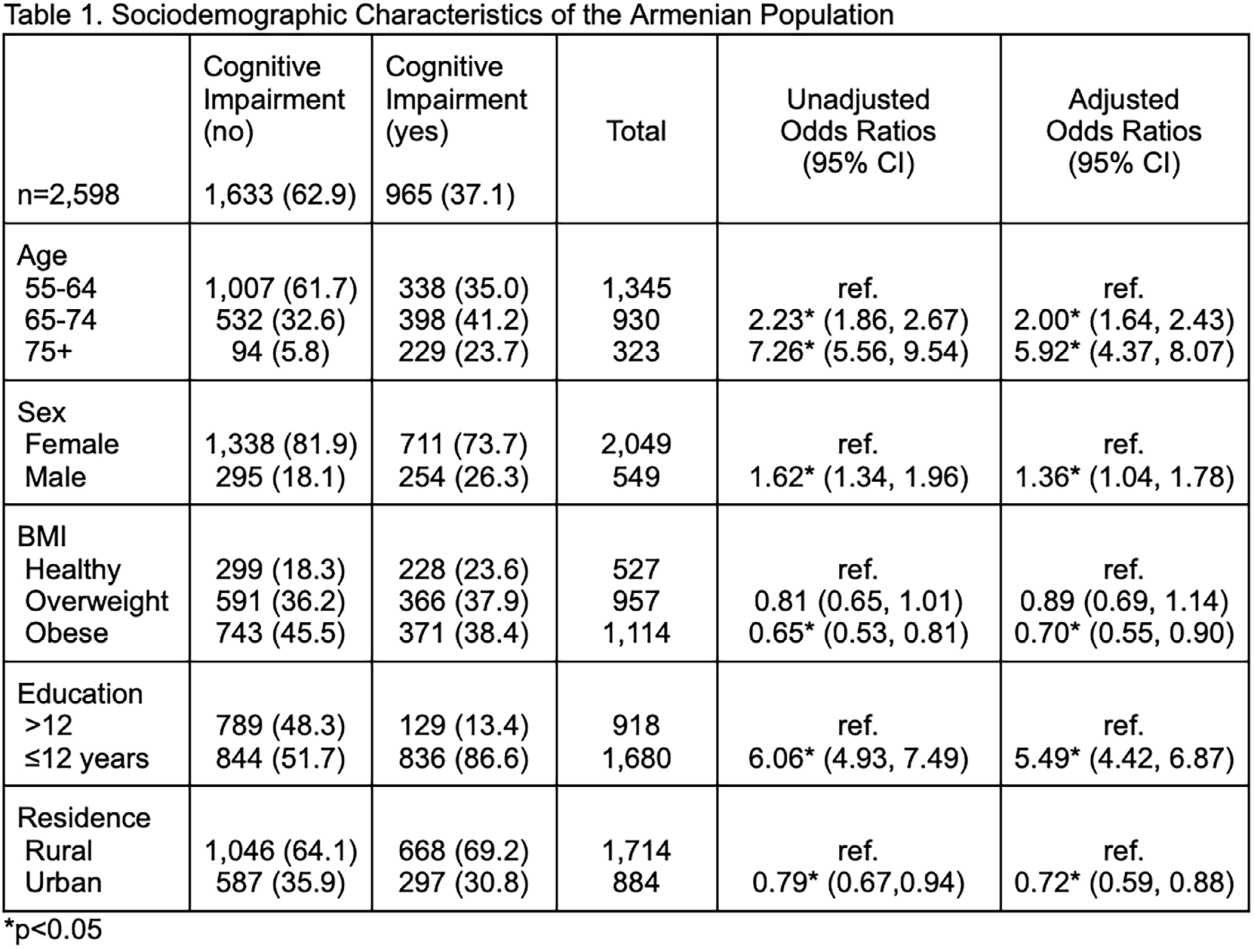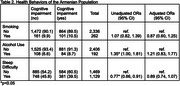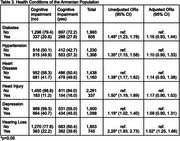# Understanding Cognitive Decline in Armenia’s Aging Population: A Nationwide Screening Study

**DOI:** 10.1002/alz70861_108717

**Published:** 2025-12-23

**Authors:** Pateel Jivalagian, Arina Megerdichian, Joan K. Monin, Armen Moughamian, Marianna Movsisyan, Abraham Hambardzumian, Jane Mahakian

**Affiliations:** ^1^ Yale University School of Public Health, New Haven, CT USA; ^2^ Alzheimer's Care Armenia, Yerevan Armenia; ^3^ University of Southern California, Los Angeles, CA USA; ^4^ Yale School of Public Health, New Haven, CT USA; ^5^ Ray Dolby Brain Health Center, Sutter Health, San Francisco, CA USA; ^6^ Alzheimer's Care Armenia, San Clemente, CA USA

## Abstract

**Background:**

Amid developing health infrastructure, the Republic of Armenia’s older adult population faces increasing concerns for cognitive impairment (CI) and subsequent dementia. The landlocked, post‐Soviet country presents a unique opportunity to study a population that has been genetically isolated with a history of stressors like generational trauma and a high burden of vascular disease. The objective of this study is to contextualize the biological and environmental risk factors associated with CI in the severely understudied Armenian population.

**Method:**

This is a population‐based, secondary analysis. Early detection cognitive screenings were conducted across 8 provinces by Alzheimer’s Care Armenia from 2022‐2023. The sample consisted of 2,598 older adults (55 years and older). The Montreal Cognitive Assessment (MoCA) was administered to assess CI. CI was dichotomized into no CI or any level of CI. Age was categorized into ten‐year intervals. Health behaviors and conditions were self‐reported. Descriptive statistics and multivariable logistic regression analysis were performed to understand population‐level trends.

**Result:**

CI was observed in 37.1% of total participants: aged 55–64 (35.1%), 65–74 years (41.2%), and 75+ (23.7%). Individuals aged 65‐74 and 75+ had increasingly higher odds of CI [AOR=2.00(1.64, 2.43); 5.92(4.37, 8.07), respectively] compared to younger individuals aged 55‐64. Males had higher odds of CI [AOR=1.36(1.04, 1.78)]. Urban residence served as a protective factor for developing CI [AOR=0.72(0.59, 0.88)]. Obese BMI level was also protective [AOR=0.70(0.55, 0.90)]. Hearing loss was significantly associated with CI [AOR=1.52(1.25, 1.86)]. These results were significant (*p* <0.05).

**Conclusion:**

CI presents an emerging problem in developing countries, like Armenia, and requires engagement with the Ministry of Health and local stakeholders to provide culturally tailored preventive care. Establishing a national brain health registry in Armenia is vital to guide research efforts, design tailored interventions, structure public health planning, and contribute to global brain health knowledge. Future research should explore the contribution of individual risk factors to CI in the Armenian population, such as a potential genetic predisposition to hearing loss.